# The role of next-generation sequencing in hematologic malignancies

**DOI:** 10.1007/s44313-024-00010-0

**Published:** 2024-03-06

**Authors:** Young-Uk Cho

**Affiliations:** Department of Laboratory Medicine, Asan Medical Center, University of Ulsan College of Medicine, 88, Olympic-Ro 43-Gil, Songpa-Gu, Seoul, 05505 Korea

**Keywords:** Next-generation sequencing, Leukemia, Diagnosis, Prognosis, Monitoring

## Abstract

Next-generation sequencing (NGS) allows high-throughput detection of molecular changes in tumors. Over the past 15 years, NGS has rapidly evolved from a promising research tool to a core component of the clinical laboratory. Sequencing of tumor cells provides an important step in detecting somatic driver mutations that not only characterize the disease but also influence treatment decisions. For patients with hematologic malignancies, NGS has been used for accurate classification and diagnosis based on genetic alterations. The recently revised World Health Organization classification and the European LeukemiaNet recommendations for acute myeloid leukemia consider genetic abnormalities as a top priority for diagnosis, prognostication, monitoring of measurable residual disease, and treatment choice. This review aims to present the role and utility of various NGS approaches for the diagnosis, treatment, and follow-up of hemato-oncology patients.

## Introduction

Molecular genetic changes in hematologic malignancies have traditionally been detected using conventional cytogenetics, fluorescence in situ hybridization, or PCR assay. Next-generation sequencing (NGS) is a method that can detect large quantities of dominant and subclonal genetic markers at once. The ability to detect multiple aberrations in multiple samples significantly enhances cost-effectiveness and reduces turnaround time compared to the stepwise testing with a single assay. Because of this innovative feature, NGS now plays a central role in identifying the molecular characteristics of cancers. In this review, we discuss the role of NGS in the diagnosis, treatment, and monitoring of hematologic malignancies.

### Targeted NGS: DNA panels

DNA-based panels are the most widely used option for molecular characterization of patients with hematologic malignancies. Recent updates to the World Health Organization 2022 classification (WHO^2022^) have expanded the subtypes defined by genetic aberrations, making targeted NGS panel assay even more critical [1, 2].

Clonal hematopoiesis (CH) is defined as the acquisition of somatic mutations in multipotent stem/progenitor cells of healthy individuals [3]. WHO^2022^ includes new myeloid precursor lesions, clonal hematopoiesis of indeterminate potential (CHIP), and clonal cytopenia of undetermined significance (CCUS) [1]. CHIP refers to CH harboring somatic mutations of myeloid malignancy-associated genes with a variant allele frequency (VAF) of ≥ 2% in individuals without a diagnosed hematologic disorder or cytopenia [1, 4]. When CHIP is accompanied by unexplained and persistent cytopenias, it is called CCUS. Common CH driver mutations are in *DNMT3A, TET2, ASXL1, JAK2, TP53, SF3B1, PPM1D, SRSF2, ZBTB33, IDH1, IDH2, U2AF1, KRAS, NRAS, CTCF, CBL, GNB1, BRCC3, PTPN11, GNAS, BCOR,* and *BCORL1* [1].

WHO^2022^ recognizes myelodysplastic syndrome (MDS) with *SF3B1* mutation as one of the MDS with defining genetic abnormalities [1, 5]. This classification highlights the importance of multi-hit *TP53* alterations by identifying a specific category exhibiting high-risk presentation and poor outcomes [1, 6]. The Molecular International Prognostic Scoring System for MDS was recently published [7]. The adverse biomarkers included multi-hit *TP53* alterations, *FLT3* mutations, and *KMT2A* partial tandem duplication, whereas *SF3B1* mutation was associated with favorable outcomes. The 16 genes with significant prognostic value were *TP53, KMT2A, FLT3, SF3B1, NPM1, RUNX1, NRAS, ETV6, IDH2, CBL, EZH2, U2AF1, SRSF2, DNMT3A, ASXL1*, and *KRAS*.

WHO^2022^ expanded the genetic mutations that define specific acute myeloid leukemia (AML) groups. *NPM1* and *CEBPA* remain AML-defining mutations. Of note, WHO^2022^ currently describes *CEBPA*-mutant AML as an entity with biallelic *CEBPA* mutations or monoallelic in-frame basic leucine zipper region (bZIP) mutations in the gene. This reflects that in-frame bZIP mutations in *CEBPA* have distinct clinical and molecular characteristics, such as younger age, enhanced co-mutation of *GATA2* and *NPM1*, and better response and improved survival [8, 9]. The term AML with myelodysplasia-related changes has been abandoned in the new system. Instead, WHO^2022^ introduced cytogenetic and molecular abnormalities associated with secondary tumor characteristics and subsequent poor prognosis. WHO^2022^ defines this category as “AML myelodysplasia-related (MR)” and includes a set of eight genes (*ASXL1, BCOR, EZH2, SF3B1, SRSF2, STAG2, U2AF1,* and *ZRSR2*) for AML-MR diagnosis [1].

Recent advances in molecular analysis have led to the recognition that more patients with myeloid malignancies than ever before have a germline predisposition [10]. This can impact patient care, such as donor selection for allogeneic stem cell transplantation, decisions regarding appropriate conditioning regimens, evaluation of family members, and genetic counseling when necessary. WHO^2022^ introduced a new category called secondary myeloid neoplasms. This category includes various pathogenic/likely pathogenic germline mutations in *CEBPA, DDX41, TP53, RUNX1, ANKRD26, ETV6*, and other mutations that are components of organ dysfunction syndromes (e.g., Fanconi anemia, Down syndrome, RASopathies, etc.) [1]. Germline mutations in *DDX41* warrant separate comment. This is the most common genetic predisposition mutation in AML and MDS. AML development is thought to be associated with a second mutation in *DDX41* in a germline mutation background in the same gene. AML with *DDX41* germline mutation has unique clinical characteristics such as male predominance, onset in the eighth decade of life, low white blood cell count, and favorable response to chemotherapy, and it is known to have an overall good prognosis [11, 12].

The updated 2022 European LeukemiaNet (ELN^2022^) risk stratification for adult AML integrated accumulated knowledge of new molecular findings and clinical trial results [13]. Notable changes in ELN^2022^ compared to previous guidelines comprise the following. First, the *FLT3*-internal tandem duplication (ITD) allelic ratio (AR) is no longer included in risk stratification. Therefore, *FLT3*-ITD mutated AML patients are considered an intermediate group, regardless of whether they have high AR or *NPM1* mutations. Second, patients with AML-MR-defining gene mutations based on WHO^2022^ are currently classified as having a poor prognosis. *RUNX1* mutations that are not classified as MR in WHO^2022^ are also considered poor genetic abnormalities in ELN^2022^. Third, the favorable prognosis of *CEBPA*-mutant AML is due to in-frame mutations affecting the bZIP region, regardless of the number of mutations. Fourth, mutant *TP53* with a VAF exceeding 10% is considered an adverse risk group. Aside from the diagnosis and risk stratification of AML, identifying molecular markers for targeted therapy has direct relevance for patient care. To date, genetic mutations associated with FDA-approved targeted therapy in patients with AML include *FLT3*-ITD and tyrosine kinase domain mutations as well as *IDH1*/*IDH2* mutations [14, 15].

The diagnosis of lymphoid neoplasms is less dependent on genetic mutations than the diagnosis of myeloid neoplasms. WHO^2022^ introduced *PAX5* mutations as one of the components of a new subtype, “B-acute lymphoblastic leukemia (ALL) with other defined genetic abnormalities” [2]. Some genetic mutations are being used to support a diagnosis of certain lymphomas. Representative examples include *BRAF* mutations for hairy cell leukemia, *MYD88* or *CXCR4* mutations for lymphoplasmacytic lymphoma, *TCF3* or *ID3* mutations for EBV-negative Burkitt lymphoma, and *STAT3* or *STAT5B* mutations for T-large granular lymphocytic leukemia [2]. Several genes are recurrently mutated and considered to be driver mutations in plasma cell myeloma (PCM): *KRAS, NRAS, IRF4, MAX, HIST1H1E, RB1, EGR1, TP53, TRAF3, FAM46C, DIS3, BRAF, LTB, CYLD,* and *FGFR3* [16, 17]. Clinically important mutated genes in hematologic malignancies are summarized in Table 1.

**Table 1:** Genetic variants detectable by next-generation sequencing assay and of clinical utility in hematologic malignancies

Diseases	Mutated genes	Fusions
Myeloid malignancies	*ABL1, ANKRD26, ASXL1*^a,b^*, BCOR*^a,b^*, BCORL1*^a^*, BRCC3*^a^*, CALR*^c^*, CBL*^a^*, CEBPA*^c^*, CTCF*^a^*, DDX41*^c^*, DNMT3A*^a^*, ETNK1, ETV6*^c^*, EZH2*^b,c^*, FLT3*^c,d^*, GNAS*^a^*, GNB1*^a^*, IDH1*^a,c^*, IDH2*^a,c^*, JAK2*^a,c^*, KIT*^c^*, KMT2A*^d^*, KRAS*^a,c^*, MPL*^c^*, NPM1*^c^*, NRAS*^a,c^*, PPM1D*^a^*, PTPN11*^a,c^*, RAD21*^c^*, RUNX1*^c^*, SETBP1*^a^*, SF3B1*^a,b,c^*, SH2B3, SRSF2*^a,b,c^*, STAG2*^b,c^*, TET2*^a^*, TP53*^a,c^*, U2AF1*^a,b,c^*, WT1*^c^*, ZBTB33*^a^*, ZRSR2*^b^	*BCR::ABL1, CBFB::MYH11, DEK::NUP214, KMT2Ar, MECOMr, NUP98r, PML::RARA, RBM15::MRTFA, RUNX1::RUNX1T1,*
Lymphoid malignancies^e^	*BRAF, CXCR4, CYLD, DIS3, EGR1, FAM46C, FGFR3, HIST1H1E, ID3, IRF4, KRAS, LTB, MAX, MYD88, NRAS, PAX5, RB1, STAT3, STAT5B, TCF3, TP53, TRAF3*	*ABL1r*^f^*, ABL2r*^f^*, BCR::ABL1, CRLF2r*^f^*, CSF1Rr*^f^*, DGKHr*^f^*, DUX4r, EPORr*^f^*, ETV6::RUNX1, IGH::IL3,* other *IGHr, IL2RBr*^f^*, JAK2r*^f^*, KMT2Ar, MEF2Dr, MYCr, NTRK3r*^f^*, NUTM1r, PAX5r, PGDFRBr*^f^*, PTK2Br*^f^*, TCF3::PBX1, TCF3::HLF, TSLPr*^f^*, TYK2r*^f^*, ZNF384r*

All genes are listed alphabetically

^a^Genes commonly mutated in clonal hematopoiesis [1]

^b^Presence of mutations in these genes defines the category ‘acute myeloid leukemia myelodysplasia-related’ according to the WHO 2022 classification [1]

^c^Basic set of genes provided by the ELN guideline may be useful in a panel approach for measurable residual disease monitoring in acute myeloid leukemia [18]

^d^Reliable detection of *FLT3*-internal tandem duplication or *KMT2A*-partial tandem duplication using targeted NGS assay may require specialized bioinformatics analysis

^e^Only genetic aberrations in blood- or bone marrow-derived lymphoid malignancies were considered

^f^These genes are typically involved in gene fusions that characterize *BCR::ABL1*-like features

### Targeted NGS: RNA panels

RNA sequencing primarily focuses on gene expression and specific gene regions coded into proteins. Clinical hematology testing, however, typically employs targeted RNA-based NGS to identify a broad spectrum of fusion transcripts and breaking points based on the WHO classification of hematologic malignancies.

While WHO^2022^ retains much of the AML-defining gene fusions, there are some noteworthy changes regarding gene fusions of “AML with defining genetic abnormalities” [1]. First, AML with *BCR::ABL1* fusion is recognized as the official subtype. Second, “AML with t(9;11)(p22;q23); *KMT2A-MLLT3*” is replaced by the new term, “AML with *KMT2A* rearrangements”. This is because more than 100 *KMT2A* fusion partners have been described [19]. While not required, the identification of the fusion partner is desirable since it can provide prognostic information and enable monitoring of the treatment response. Third, “AML with *NUP98* rearrangement” is recognized as a new subtype. *NUP98* is rearranged with multiple partners, in many cases in a cryptic manner, and is generally associated with poor clinical outcomes [20, 21].

The classification of ALL based on gene fusions remains largely unchanged from previous WHO criteria. Some minor updates reflect the incorporation of additional gene fusions and refinements in the definitions of entities based on shared gene expression features [2]. First, the rare “B-ALL with *TCF3::HLF* fusion” has been added to WHO^2022^. It has been reported to be a poor prognostic group [22, 23]. Second, “B-ALL with *BCR::ABL1*-like features” is now an official entity. It is characterized by clusters on gene expression profiling (GEP) with B-ALL with *BCR::ABL1* while indeed lacking *BCR::ABL1* fusion. Identification of *BCR::ABL1*-like features is challenging due to the diversity of aberrations and the requirement of a microarray method for GEP. Targeted RNA sequencing can be an alternative diagnostic tool for this subgroup because this entity is commonly associated with gene fusions involving *CRLF2, JAK2, ABL1, PDGFRB, ABL2, EPOR, PTK2B, CSF1R, DGKH, IL2RB, NTRK3, TSLP,* and *TYK2* [22, 24]. Similarly, advances in diagnostic methodologies have allowed the identification of a new entity, “B-ALL with *ETV6::RUNX1*-like features” [22, 25]. Third, “B-ALL with other defined genetic abnormalities” includes *MYC, DUX4, MEF2D, ZNF384,* or *NUTM1* rearrangements, as well as *PAX5* alterations [22]. Unlike B-ALL, there is as yet not sufficient evidence to establish genetically defined subtypes of T-ALL with clinical relevance. In PCM, the large majority of gene fusions affect *IGH* and, in a small number, *MYC* [17]. Clinically important fusion genes in hematologic malignancies are summarized in Table 1.

### Beyond targeted NGS

There have been several studies that have employed non-targeted NGS for genomic profiling of patients with hematologic malignancies. A representative work is an application of whole-genome sequencing (WGS) on patients with AML. WGS detected all recurrent translocations and copy-number alterations that had been identified by cytogenetic analysis. Prospective sequencing of samples provided new genetic information in a quarter of patients, which changed the risk category in a subset of patients [26]. The other study applied WGS to patients with childhood ALL. They showed that WGS detected subtype-defining genetic abnormalities in almost all patients and identified novel genetic variants including fusions involving genes in the MAP kinase pathway [27]. Another study applied whole transcriptome sequencing (WTS) on patients with AML and MDS. WTS identified fusion genes in 37% of AML and 3% of MDS patients. In AML, half of all detected fusions were entity-defining rearrangements. Interestingly, 41% of the fusions found in AML patients and 88% of the fusions found in MDS patients were novel fusions that had not been previously reported [28]. Thus, WGS and WTS highlight the complexity of molecular genetic features of hematologic malignancies in addition to revealing molecular markers that have been hidden due to the limitations of targeted NGS. However, their high cost and the requirement for complicated bioinformatics make them not yet available as a daily test in most clinical laboratories.

### Monitoring of disease: NGS-based measurable residual disease (MRD) and chimerism assay

MRD is considered one of the key indicators for the evaluation of treatment responses in patients with hematologic malignancies, especially acute leukemia. MRD has conventionally been detected by real-time quantitative PCR (RQ-PCR) or flow cytometry [29]. In lymphoid malignancies such as ALL or PCM, the mainstream platform employs NGS assay to measure the clonality of *IGH* and *TCR* gene rearrangements [30, 31]. NGS-based MRD is highly concordant with RQ-PCR and can be an alternative in the front line of MRD evaluation in forthcoming MRD-based protocols for patients with pediatric ALL [32]. In myeloid malignancies, the target for NGS-based MRD assay should be tumor-associated somatic mutations. There have been several studies demonstrating the clinical relevance of NGS-based MRD monitoring in patients with AML [33–35]. The revised ELN guideline recommends error-corrected NGS with unique molecular identifiers as one of the MRD techniques to obtain a limit of detection of 10^–3^ or lower in AML [18]. This guideline also provides a basic set of genes that covers a large proportion of patients with AML: *CALR, CEBPA, DDX41, ETV6, EZH2, FLT3, IDH1, IDH2, JAK2, KIT, KRAS, MPL, NPM1, NRAS, PTPN11, RAD21, RUNX1, SF3B1, SRSF2, STAG2, TP53, U2AF1,* and *WT1* (Table 1). Of note, germline mutations and mutations associated with age-related CH (so-called DTA) should be excluded from MRD analysis. It is expected that standardization and application guidelines for NGS-based MRD assay for AML or MDS will be established in order to facilitate the clinical use of NGS-based MRD assay in the field of myeloid malignancies in the foreseeable future. Recently, an NGS-based chimerism assay using a panel of single-nucleotide polymorphisms was developed and validated. It exhibited good concordance with the conventional method and provided accurate and sensitive monitoring of the engraftment state and detection of early relapse [36, 37].

## Conclusions

High-throughput technologies such as NGS now play a pivotal role in characterizing the molecular features of hematologic malignancies throughout their clinical course. The role of NGS assay ranges from diagnosis and prognostication to post-treatment monitoring. Expanding the utility of NGS assay will require increasingly more resources, including higher-specification analyzers and bioinformatics support, but clinical laboratories should continue their efforts to implement and apply this innovative technology into routine practice.

## Data Availability

No datasets were generated or analysed during the current study.
